# T Cell Polarization toward T_H_2/T_FH_2 and T_H_17/T_FH_17 in Patients with IgG4-Related Disease

**DOI:** 10.3389/fimmu.2017.00235

**Published:** 2017-03-13

**Authors:** Aurélie Grados, Mikael Ebbo, Christelle Piperoglou, Matthieu Groh, Alexis Regent, Maxime Samson, Benjamin Terrier, Anderson Loundou, Nathalie Morel, Sylvain Audia, François Maurier, Julie Graveleau, Mohamed Hamidou, Amandine Forestier, Sylvain Palat, Emmanuelle Bernit, Bernard Bonotte, Catherine Farnarier, Jean-Robert Harlé, Nathalie Costedoat-Chalumeau, Frédéric Vély, Nicolas Schleinitz

**Affiliations:** ^1^AP-HM, Service de Médecine Interne, Hôpital de la Timone, Marseille, France; ^2^Aix-Marseille Université, CNRS, INSERM, Centre d’Immunologie de Marseille-Luminy, Marseille, France; ^3^AP-HM, Service d’Immunologie, Hôpital de la Conception, Marseille, France; ^4^Service de Médecine Interne, CHU Cochin, AP-HP, Paris, France; ^5^Université Paris Descartes, Paris, France; ^6^Service de Médecine Interne, CHU le Bocage, Dijon, France; ^7^Université de Bourgogne, Dijon, France; ^8^AP-HM, Unité d’Aide Méthodologique, Aix-Marseille Université, Marseille, France; ^9^Service de Médecine Interne, Hôpital Sainte Blandine, Metz, France; ^10^Service de Médecine Interne, CHU de Nantes, Nantes, France; ^11^Université de Nantes, Nantes, France; ^12^Service d’Immunologie clinique, Groupe hospitalier mutualiste, Grenoble, France; ^13^Service de Médecine Interne, CHU Dupuytren, Limoges, France

**Keywords:** IgG4-related disease, T helper cells, T follicular helper cells, plasmablasts, Sjögren’s syndrome

## Abstract

IgG4-related disease (IgG4-RD) is a fibro-inflammatory disorder involving virtually every organ with a risk of organ dysfunction. Despite recent studies regarding B cell and T cell compartments, the disease’s pathophysiology remains poorly understood. We examined and characterized subsets of circulating lymphocytes in untreated patients with active IgG4-RD. Twenty-eight consecutive patients with biopsy-proven IgG4-RD were included in a prospective, multicentric study. Lymphocytes’ subsets were analyzed by flow cytometry, with analysis of T_H_1/T_H_2/T_H_17, T_FH_ cells, and cytokine release by peripheral blood mononuclear cells. Results were compared to healthy controls and to patients with primary Sjögren’s syndrome. Patients with IgG4-RD showed an increase of circulating T regulatory, T_H_2, T_H_17, and CD4^+^CXCR5^+^PD1^+^ T_FH_ cell subsets. Accordingly, increased levels of IL-10 and IL-4 were measured in IgG-RD patients. T_FH_ increase was characterized by the specific expansion of T_FH_2 (CCR6^−^CXCR3^−^), and to a lesser extent of T_FH_17 (CCR6^+^CXCR3^−^) cells. Interestingly, CD4^+^CXCR5^+^PD1^+^ T_FH_ cells normalized under treatment. IgG4-RD is characterized by a shift of circulating T cells toward a T_H_2/T_FH_2 and T_H_17/T_FH_17 polarization. This immunological imbalance might be implicated in the disease’s pathophysiology. Treatment regimens targeting such T cells warrant further evaluation.

## Introduction

IgG4-related disease (IgG4-RD) is a newly recognized condition characterized by mass-forming lesions involving various tissues (1). The disease more frequently affects males over 50 years of age (2–4). The unifying finding of the disease is the pathological lesion characterized by dense lymphoplasmacytic infiltrates mainly consisting of CD4^+^ T cells and numerous IgG4^+^ plasma cells associated with fibrosis (5). Tertiary germinal center formation is also frequently observed in diseased tissues.

Mikulicz’s disease, otherwise considered as a disease manifestation of primary Sjögren’s syndrome (pSS) (6) is also part of the spectrum of IgG4-RD. It has now been clearly demonstrated that despite common features shared by both diseases, clinical, biological, pathological, and immunohistological findings differ between pSS and IgG4-RD (7). Yet, in daily practice, pSS remains a frequent differential diagnosis of IgG4-RD.

Despite ongoing genetic and mechanistic studies, the pathogenesis of IgG4-RD remains poorly understood. Rather than a vector of tissue damage, IgG4 is considered to be a marker of the disease. Yet, elevated levels of serum IgG4 have also been reported in other conditions, and approximately 30% of patients with biopsy-proven IgG4-RD have normal serum levels of IgG4 (8). Hence, the identification of novel biomarkers is a timely topic in the field of IgG4-RD.

Besides studies regarding B cells and immunoglobulin biology, careful attention is currently being paid on the role of T cells in the disease. Conflicting results have been reported regarding the polarization of T helper (T_H_) cells in IgG4-RD, especially in patients with pancreatic involvement and Mikulicz’s disease (9, 10). Because regulatory T cells (Treg) are a source of IL-10 and TGF-β (i.e., key cytokines involved, respectively, in the differentiation of IgG4-producing B cells and in the genesis of fibrosis), the roles of these cells have also been investigated in a few preliminary studies regarding IgG4-related hepatic and pancreatic involvements (11–13) More recently, T follicular helper cells (T_FH_), an important cell subset involved in the development of germinal centers as well as in antibody production, have also been studied in a small series of IgG4-RD patients (14) suggesting changes of this compartment in the disease. Yet, these results need to be confirmed in a new and larger cohort, including patients with other than salivary and lachrymal-restricted involvements, and with a more precise phenotypic definition of T_FH_ subsets, especially concerning PD1 molecule expression.

In this study, we investigated circulating lymphocytes in untreated patients with IgG4-RD, with a focus on T_H_1/T_H_2/T_H_17 balance and PD1^+^ T_FH_ cells, and compared them to pSS patients.

## Patients and Methods

### Study Subjects

Patients with IgG4-RD were identified according to the Comprehensive Diagnosis Criteria (CDC) for IgG4-RD or to the International Consensus Diagnostic Criteria (ICDC) for autoimmune pancreatitis (15). Patients with pSS were identified according to the 2002 American-European Consensus Group criteria. Patients with either IgG4-RD or pSS who received steroids or disease-modifying antirheumatic drugs within 3 months prior to study entry or who received rituximab within 6 months prior to enrollment were excluded from the study. Written informed consent was obtained from all patients with IgG4-RD or pSS as well as healthy controls (HC) prospectively included in the study. The study was approved by the local ethics committee [CPP Marseille I (Comité de Protection des Personnes Marseille I)].

### Immunophenotyping by Flow Cytometry

Lymphocyte populations (total lymphocytes, T cells, CD4^+^ T cells, CD8^+^ T cells, B cells, and natural killer (NK) cells) were quantified with 6-Color BD Multitest and BD Trucount Technologies (Becton Dickinson, Le Pont de Claix, France) according to the manufacturer’s instructions.

MSL (Eurobio) density centrifugation was used to separate peripheral blood mononuclear cells (PBMCs) immediately after blood sample collection. The following antibodies were used: APC-H7 anti-CD45 or V500-anti-CD45, Amcyan-anti-CD3 or APC-H7-anti-CD3, APC-H7-anti-CD4 or PE-anti-CD4, PerCP-Cy5.5-anti-CD8 or APC-anti-CD8, APC-H7-anti-CD20, V450-anti-CD45RA or PE-Cy5-anti-CD45RA, PE-Cy7-anti-CD45RO, Alexa Fluor 488-anti-CXCR5, Alexa Fluor 647-anti-PD1, PE-anti-CCR6, PE-Cy7-anti-CXCR3, APC-anti-CD25 (BD Biosciences), PerCP-Cy5.5-anti-CD19, FITC-anti-CD62L (Beckman Coulter), or isotype-matched controls for 30 min.

T_FH_ cells were defined as CD4^+^CD45RA^−^CXCR5^+^PD1^+^. Three T_FH_ subsets were defined according to the expression of CCR6 and CXCR3, as follows: CCR6^−^CXCR3^+^ T_FH_1 cells, CCR6^−^CXCR3^−^ T_FH_2 cells, and CCR6^+^CXCR3^−^ T_FH_17 cells. Plasmablasts were defined as CD19^+^CD27^high^CD38^high^, naïve T cells as CD4^+^CD45RA^high^, memory T cells as CD4^+^CD45RO^+^, and NK cells as CD3^−^CD56^+^.

For intracellular markers, fixed and permeabilized cells were stained using Alexa Fluor 488-anti-FoxP3 (BD Biosciences) to analyze T regulatory cells defined as CD4^+^FoxP3^+^; PE-anti-IL-17, FITC-anti-IFNγ, and APC-anti-IL-4 (BD Bioscience) were used to characterize the functionally polarized CD4^+^ T cell subsets after stimulation with PMA-ionomycin for 5 h according to the manufacturers’ instructions. The cells were washed with phosphate-buffered saline and then analyzed on a BD FACS Canto II.

### Analysis of Cytokine Production

Levels of IL-4, IL-10, IL-17, and IFNγ in the supernatant of PBMCs following a 24-h stimulation with PMA-ionomycin were assessed using a multiplexed bead-based immunoassay (CBA^®^ kit, BD Biosciences) following the manufacturers’ protocol.

### Statistical Analysis

Continuous variables are shown as median ± SD. Multiple group comparisons were analyzed using the Kruskal–Wallis test and the Mann–Whitney *U*-test was used for comparison between two groups. Correlations were analyzed using Spearman’s correlation coefficient. The Wilcoxon test was used to analyze the changes in values over time. Statistical analyses were performed with Prism 6 (GraphPad Software, San Diego, CA, USA).

For multiple testing, adjusted *p*-values were calculated using the false discovery rate procedure with the PROC MULTTEST statement (16). Statistical tests for multiple testing were made with the SAS 9.4 software.

## Results

### Patients’ Characteristics

Patients with IgG4-RD included in this study fulfilled the CDC (*n* = 27) or the ICDC criteria (*n* = 1). Clinical, biological, and pathological characteristics of these patients are reported in Tables 1 and 2. Mean age was 63.6 ± 15, 57 ± 13, and 59 ± 17.3 years in patients with IgG4-RD, pSS (*n* = 21), and HC (*n* = 28), respectively. Male gender was overrepresented in patients with IgG4-RD as compared to pSS and HC (85.5% versus 23 and 57%).

**Table 1:** **General characteristics of patients with IgG4-related disease (IgG4-RD)**.

*n*	G	Age	Organ involvement	IgG4 g/l	IgG4-RD RI	Status
1	M	65	Parotid, LN	0.59	6	Relapse
2	M	66	RPF, LN, lung	4.74	12	First flare
3	M	66	Meninges, aorta	3.61	7	Relapse
4	M	57	Pancreas	6.3	6	First flare
5	M	78	Kidney, LN, lung, pancreas	21.9	13	Relapse
6	F	63	SMG, LN	1.56	9	First flare
7	F	80	RPF, lung, thyroid	0.49	6	First flare
8	M	47	RPF, pancreas, lung	1.34	2	Relapse
9	M	86	Lung, LN, RPF	15.7	10	Relapse
10	M	76	Bone, LN	27.5	9	First flare
11	M	66	RPF, lung, kidney	2.56	12	First flare
12	M	37	Kidney, lung, pancreas, prostate, testis, LN	18.5	15	Relapse
13	M	31	Orbit, lung, LN, pancreas, SMG	15.5	12	Relapse
14	M	80	Mesenteritis	3.16	4	First flare
15	F	39	Orbit	1.04	6	First flare
16	M	74	Kidney, pancreas, LN	3.66	12	Relapse
17	M	63	RPF	7.4	6	First flare
18	M	63	LN	10.9	6	First flare
19	M	52	Pancreas, LN, bile duct	10.2	9	Relapse
20	M	78	Pancreas	4.5	3	First flare
21	M	57	Bile duct	1.1	3	First flare
22	M	67	Lung, SMG, LN	5.84	9	First flare
23	M	82	Kidney, SMG, lung, LN	36.7	15	Relapse
24	M	43	LN, pancreas, bile duct	22.4	5	First flare
25	M	79	LN, pancreas	3.2	3	First flare
26	M	77	Pancreas, skin	16.4	3	First flare
27	F	46	Pancreas, liver, lung, LN	3,59	12	First flare
28	M	63	Dacryoadenitis, LN, pancreas	12,3	12	First flare

*F, female; G, gender; LN, lymph nodes; M, male; n, number; RI, responder index; RPF, retroperitoneal fibrosis; SMG, submandibular glands*.

**Table 2:** **Pathological characteristics of patients with IgG4-RD**.

P	Tissue	DLPI	SF	OP	Eo	IgG4/IgG IgG4/CD138^a^	IgG4/HPF
1	Parotid	x	x			50%/IgG	>50
2	RPF	x	x			>50%/CD138	80
3	Meninges	x	x		x	70%/CD138	60
4	Pancreas	x				50%/CD138	45
5	Kidney	x	x			ND	30
6	SMG	x	x	x		>40%/IgG	20
7	Lung	x	x			ND	15
8	Pancreas, bile duct	x	x			>40%/CD138	10
9	RPF	x	x			50%/CD138	60
10	Lymph node	x			x	80%/CD138	90
11	Kidney	x	x	x	x	50%/IgG	ND
12	Kidney	x	x		x	50%/CD138	30
13	SMG, pancreas	x	x	x		90%/IgG	ND
14	Mesenteritis	x	x			50%/CD138	30
15	Orbit	x	x		x	ND	>10
16	Kidney	x	x			>50%/CD138	40
17	Pancreas	x				ND	40
18	Lymph node	x				ND	100
19	Bile duct	x	x			ND	>10
20	Pancreas	x	x			>40%/CD138	50
21	Bile duct	x	x		x	ND	>10
22	Lymph node	x			x	80%/CD138	40
23	Lymph node	x			x	>50%/IgG	30
24	Pancreas	x	x			ND	>100
25	Pancreas	x	x			ND	80
26	Pancreas, skin	x	x			>90%/CD138	>40
27	Liver	x	x		x	0	0
28	SMG	x	x			50%/IgG	ND

*^a^IgG4 plasmocyte ratio is given either from IgG^+^ or total CD138^+^ plasmocytes*.

*DLPI, diffuse lymphoplasmacytic infiltrate; Eo, eosinophils; HPF, high power field; ND, not determined; OP, obliterative phlebitis; RPF, retroperitoneal fibrosis; SF, storiform fibrosis; SMG, submandibular gland*.

IgG4 levels were >1.35 g/l (mean 7.9 ± 6.1 g/l) in 82% of patients with IgG4-RD, median IgE level was 627 ± 1,092 UI/l and median eosinophil count 443 ± 499 cells/μl (19% of patients had >500 cells/μl) in IgG4-RD patients. Most patients with IgG4-RD (67%) were analyzed at the first flare of the disease. Organ involvement at the time of analysis included lymph nodes (*n* = 17), lung (*n* = 10), pancreas (*n* = 8), retroperitoneal fibrosis (*n* = 5), kidney (*n* = 5), salivary glands (*n* = 4), orbit (*n* = 3), and bile ducts (*n* = 2), and 64% of patients with IgG4-RD presented with ≥2 organs involved (Table 1).

### Lymphocyte Subsets in Patients with IgG4-RD

**Figure 1:**
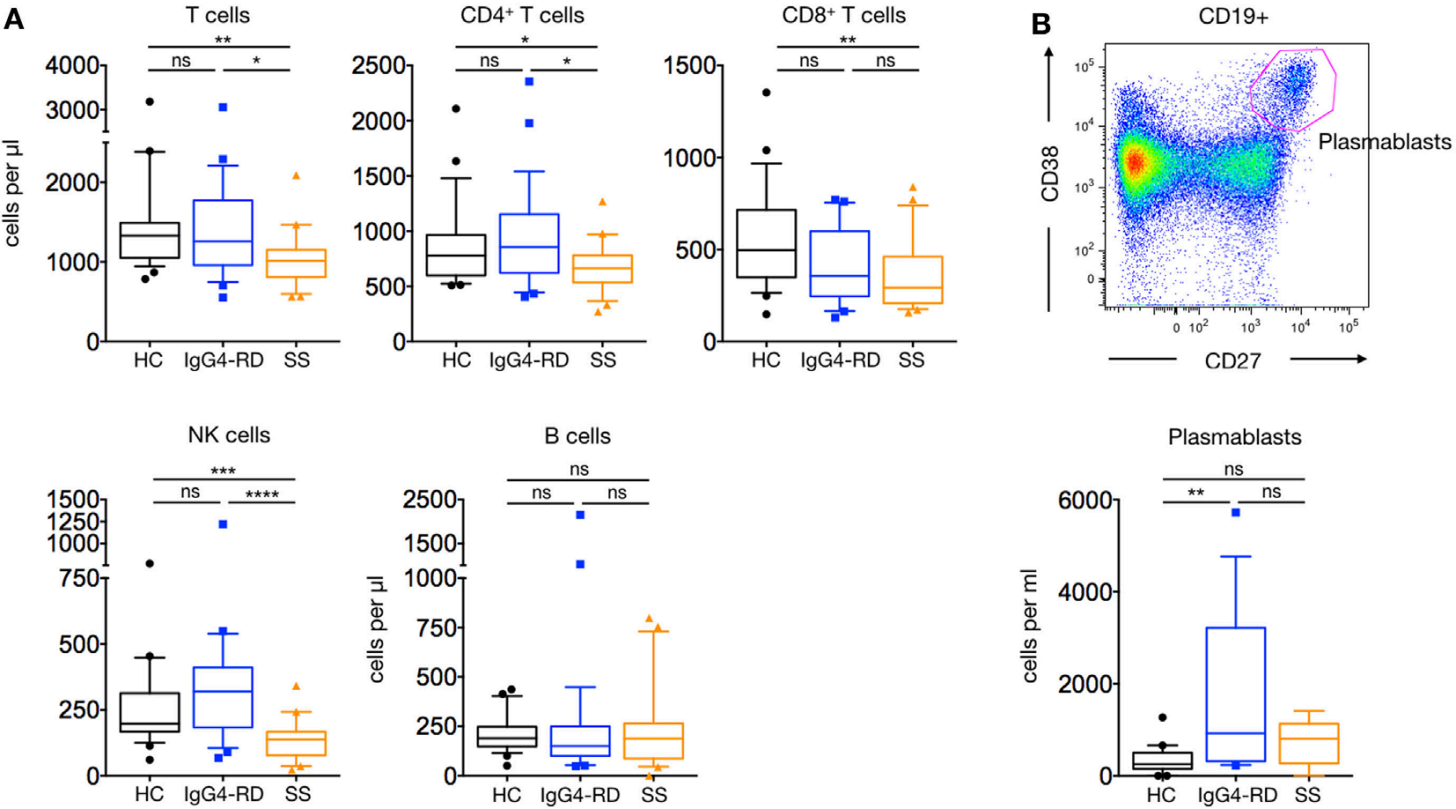
**Lymphocyte subsets in IgG4-related disease (IgG4-RD) patients**. IgG4-RD patients (blue lines) are compared to healthy controls (black lines) and Sjögren syndrome patients (orange lines). Peripheral blood T, B, and NK cells counts are shown **(A)**. A representative staining used to detect plasmablasts (upper panel) and their cell counts in the three groups (lower panel) are shown **(B)**. Bars show the median and the 10th and 90th percentiles. If the Kruskal–Wallis test is significant, groupwise comparisons are performed by the Mann–Whitney *U*-test. *p* is considered significant when <0.05; *p < 0.05; **p < 0.01; ***p < 0.001; ****p < 0.0001.

Analysis of patients with IgG4-RD showed no difference in the number and proportion of CD4^+^, CD8^+^ T cell subsets, and NK cells compared to HC (Figure 1A). Interestingly, both the number (1,685 ± 418 cells/μl versus 351 ± 66 cells/μl; *p* = 0.001) and proportion of circulating plasmablast cells were increased in patients with IgG4-RD compared to HC, although B cell counts were similar within the three groups (Figure 1B).

**Figure 2:**
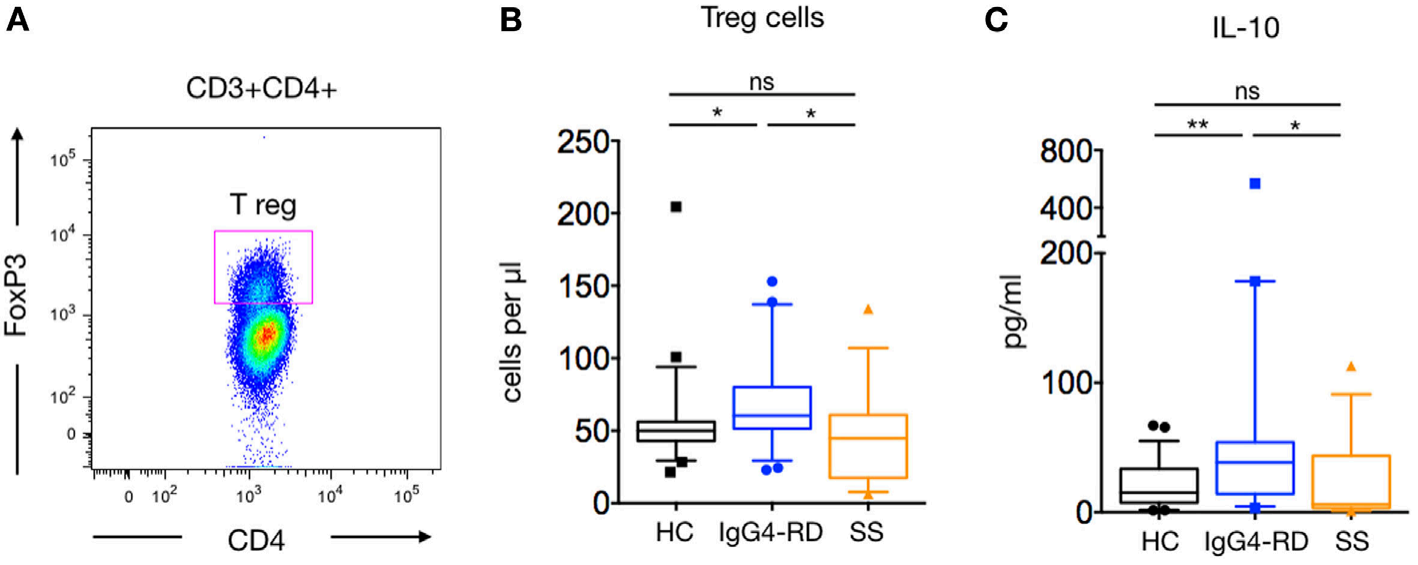
**Regulatory T cells (Treg) cells and IL-10 release in IgG4-related disease (IgG4-RD) patients**. A representative staining of CD4^+^FoxP3 Treg cells is shown **(A)**. Peripheral blood Treg cells counts in IgG4-RD patients (blue lines), healthy controls (HC) (black lines), and Sjögren syndrome patients (orange lines) **(B)**. IL-10 release by PBMC after PMA-ionomycin stimulation is shown in IgG4-RD patients (blue lines), HC (black lines), and Sjögren syndrome patients (orange lines) **(C)**. Bars show the median and the 10th and 90th percentiles. If the Kruskal–Wallis test is significant, groupwise comparisons are performed by the Mann–Whitney *U*-test. *p* is considered significant when <0.05; *p < 0.05; **p < 0.01.

CD4^+^Foxp3^+^ Treg were significantly increased in patients with IgG4-RD compared to HC and pSS patients (71.11 ± 7.3 cells/μl versus 57 ± 6.8 and 46.8 ± 8.3 cells/μl; *p* = 0.01 and *p* = 0.02, respectively) (Figures 2A,B). As Treg cells produce IL-10, the ability of PBMCs to produce IL-10 was evaluated following PMA-ionomycin stimulation. A significantly higher amount of IL-10 was detected in supernatants of stimulated PBMCs from patients with IgG4-RD compared to HC and pSS patients (70.6 ± 29.1 pg/ml versus 20.8 ± 3.8 and 24.2 ± 9.2 pg/ml; *p* = 0.009 and *p* = 0.03, respectively) (Figure 2C).

### T_H_1, T_H_2, and T_H_17 Cell Distribution in Patients with IgG4-RD

**Figure 3:**
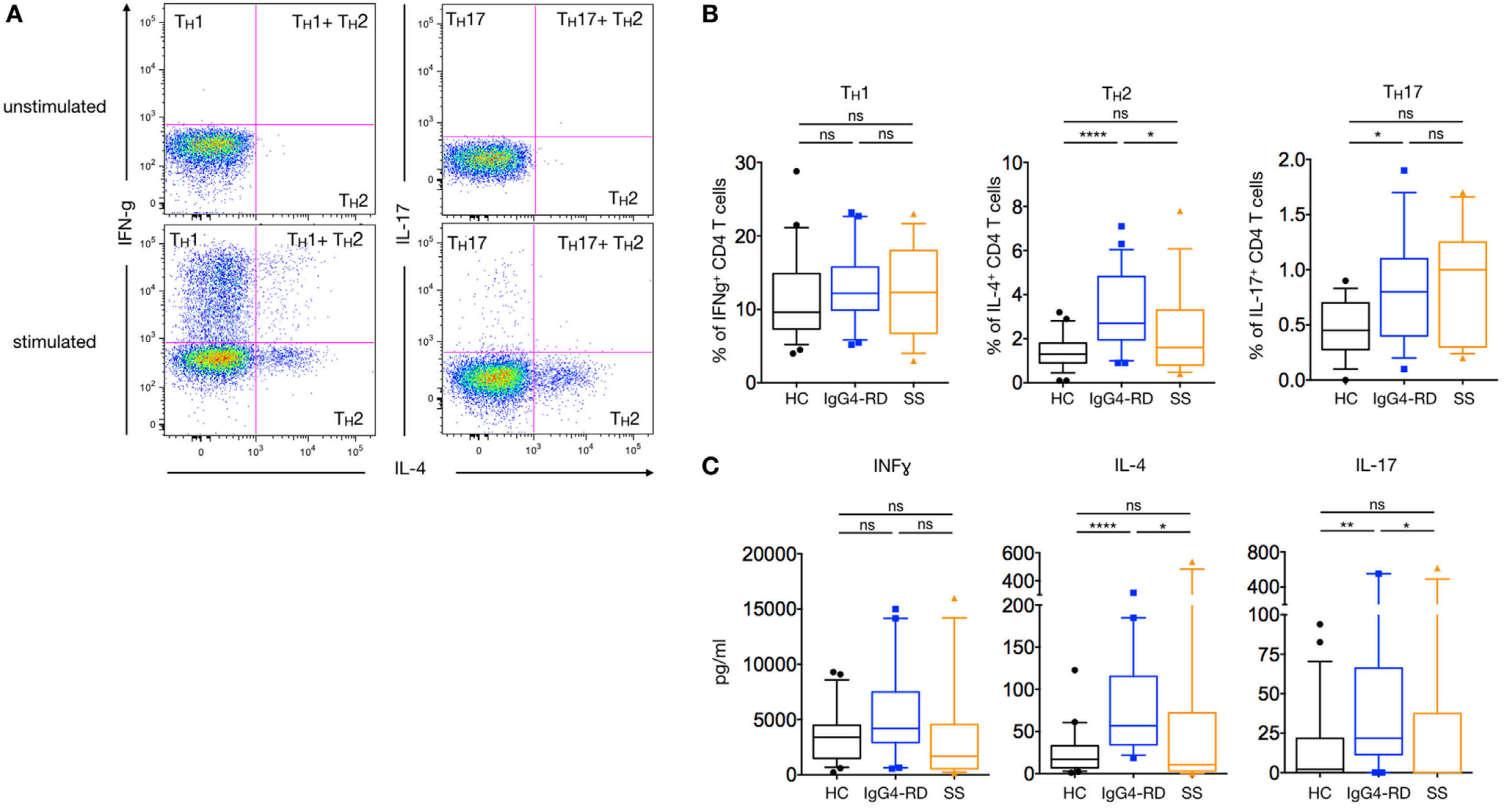
**T_H_1/T_H_2/T_H_17 profile and cytokine production in IgG4-related disease (IgG4-RD) patients**. A representative staining showing interferon gamma (T_H_1 cells)-, IL-4 (T_H_2)-, and IL-17 (T_H_17)-producing CD4^+^ T cells without stimulation (upper dot-plots) or after PMA-ionomycin treatment (lower dot-plots) **(A)**. Percentages of T_H_1, T_H_2, and T_H_17 cells after PMA-ionomycin stimulation in IgG4-RD patients (blue lines), healthy controls (HC) (black lines), and Sjögren syndrome patients (orange lines) **(B)**. Cytokine secretion by PBMC after PMA-ionomycin stimulation in IgG4-RD patients (blue lines), HC (black lines), and Sjögren syndrome patients (orange lines) **(C)**. Bars show the median and the 10th and 90th percentiles. If the Kruskal–Wallis test is significant, groupwise comparisons are performed by the Mann–Whitney *U*-test. *p* is considered significant when <0.05; *p < 0.05; **p < 0.01; ****p < 0.0001.

To analyze the distribution of functionally polarized CD4^+^ T cell subsets in patients with IgG4-RD, PBMCs were stimulated for 5 h using PMA-ionomycin (Figures 3A,B). The percentage of IL-4-producing CD4^+^ T cells (T_H_2) was significantly higher in patients with IgG4-RD compared to HC and pSS patients (3.4 ± 0.36% versus 1.5 ± 0.14 and 2.2 ± 0.5%; *p* < 0.0001 and *p* = 0.03, respectively). In addition, IL-17^+^-producing CD4^+^ T cells (T_H_17) were significantly higher in patients with IgG4-RD compared to HC (1.0 ± 0.17% versus 0.7 ± 0.17%; *p* = 0.02) but not to patients with pSS. Conversely, the proportion of T_H_1 IFNγ^+^ producing cells was similar in all three groups of patients.

### Cytokine Secretion Profile in Patients with IgG4-RD

In order to further confirm the trend toward a T_H_2/T_H_17 balance in IgG4-RD, PBMCs’ cytokines production following a 24-hour stimulation with PMA-ionomycin was measured using a multiplexed CBA assay. As shown in Figure 3C, higher levels of IL-4 and IL-17 were detected in patients with IgG4-RD as compared to HC and pSS patients (82.4 ± 16.8 pg/ml versus 24.1 ± 5.6 and 89.7 ± 48.1 pg/ml for IL-4; *p* < 0.0001 and *p* = 0.02, respectively; and 120.4 ± 65.4 pg/ml versus 15.5 ± 5.5 and 80 ± 49 pg/ml for IL-17; *p* = 0.006 and *p* = 0.01, respectively). No such difference was observed with levels of IFNγ.

### CD4^+^CXCR5^+^PD1^+^ T_FH_ Are Specifically Increased in Patients with IgG4-RD

**Figure 4:**
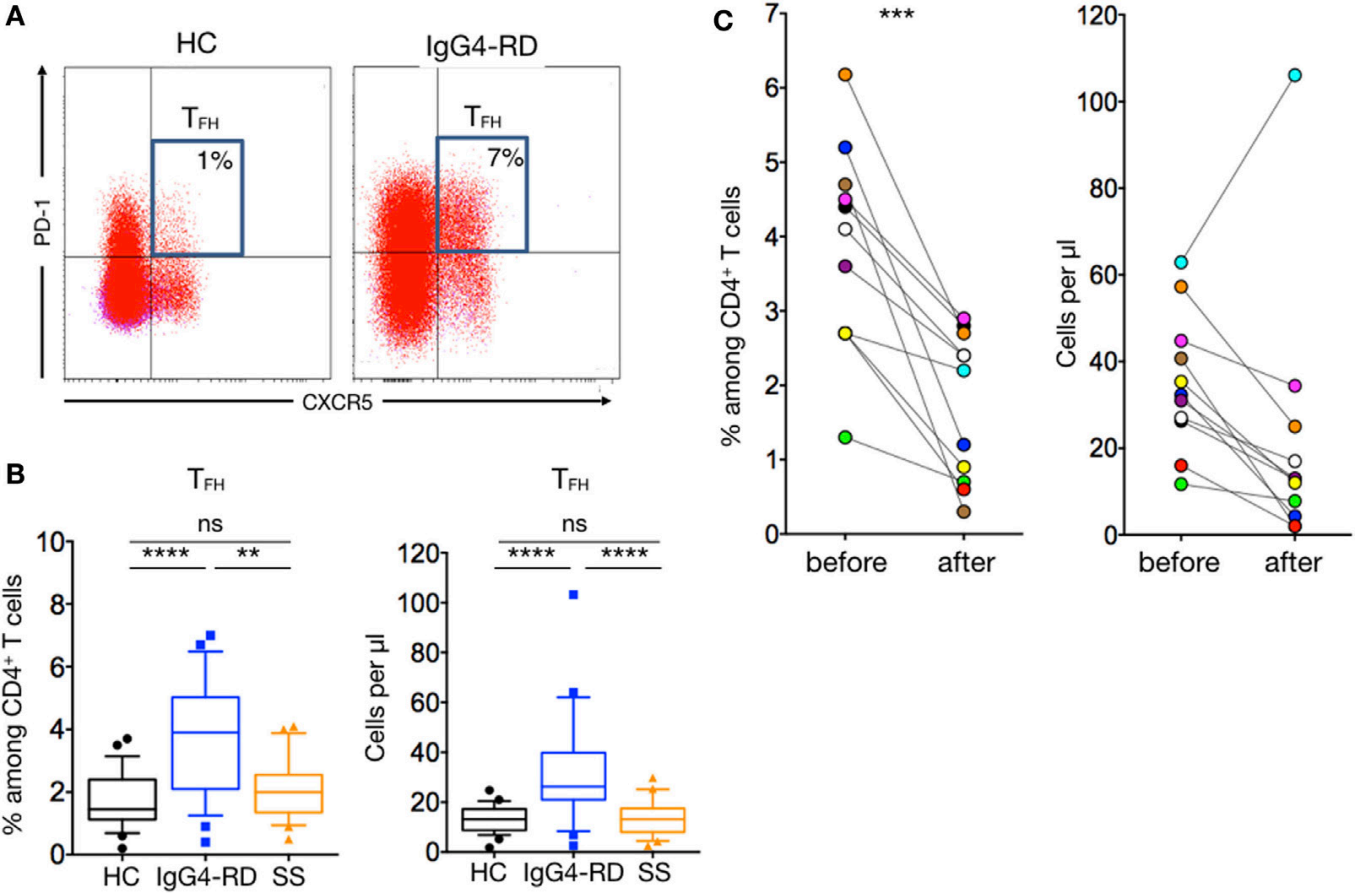
**T follicular helper cells in IgG4-related disease (IgG4-RD) patients**. Representative staining showing an expansion of CXCR5^+^PD1^+^ T_FH_ cells in untreated patients with IgG4-RD compared to an healthy individual **(A)**. CXCR5^+^PD1^+^ T_FH_ cells percentage (left panel) and absolute numbers (right panel) are higher in IgG4-RD patients (blue lines) than in healthy controls (black lines) and Sjögren syndrome patients (orange lines) **(B)**. Both T_FH_ cells percentage (left panel) and absolute numbers (right panel) decrease after treatment in patients with IgG4-RD **(C)**. Bars show the median and the 10th and 90th percentiles. If the Kruskal–Wallis test is significant, groupwise comparisons are performed by the Mann–Whitney *U*-test. *p* is considered significant when <0.05; **p < 0.01; ***p < 0.001; ****p < 0.0001.

The ectopic lymphoid structures found in tissues of patients with IgG4-RD led us to investigate for the presence of T_FH_ cells in patients’ peripheral blood. Such cells were defined as CD4^+^CXCR5^+^CD45RA^−^PD1^+^ lymphocytes (Figure 4A). This subset was significantly increased in patients with IgG4-RD when compared to HC and pSS patients in both percentages (3.7 ± 0.3% versus 1.7 ± 0.1 and 2 ± 0.2%; *p* < 0.0001 and *p* = 0.001, respectively) and numbers (35.4 ± 4.8 cells/μl versus 13.4 ± 0.9 and 13.9 ± 1.6 cells/μl; both *p* < 0.0001, respectively) (Figure 4B). We also evaluated PD1^+^ T_FH_ cell number in 11 IgG4-RD patients who were treated with steroids alone (*n* = 4), steroids plus azathioprine (*n* = 1), or steroids plus rituximab (*n* = 6). Interestingly, disease remission in these treated IgG4-RD patients was associated with reduced circulating T_FH_ cells (Figure 4C).

### T_FH_2 and T_FH_17 Are the Main T Follicular Helper Cells Expanded in Patients with IgG4-RD

**Figure 5:**
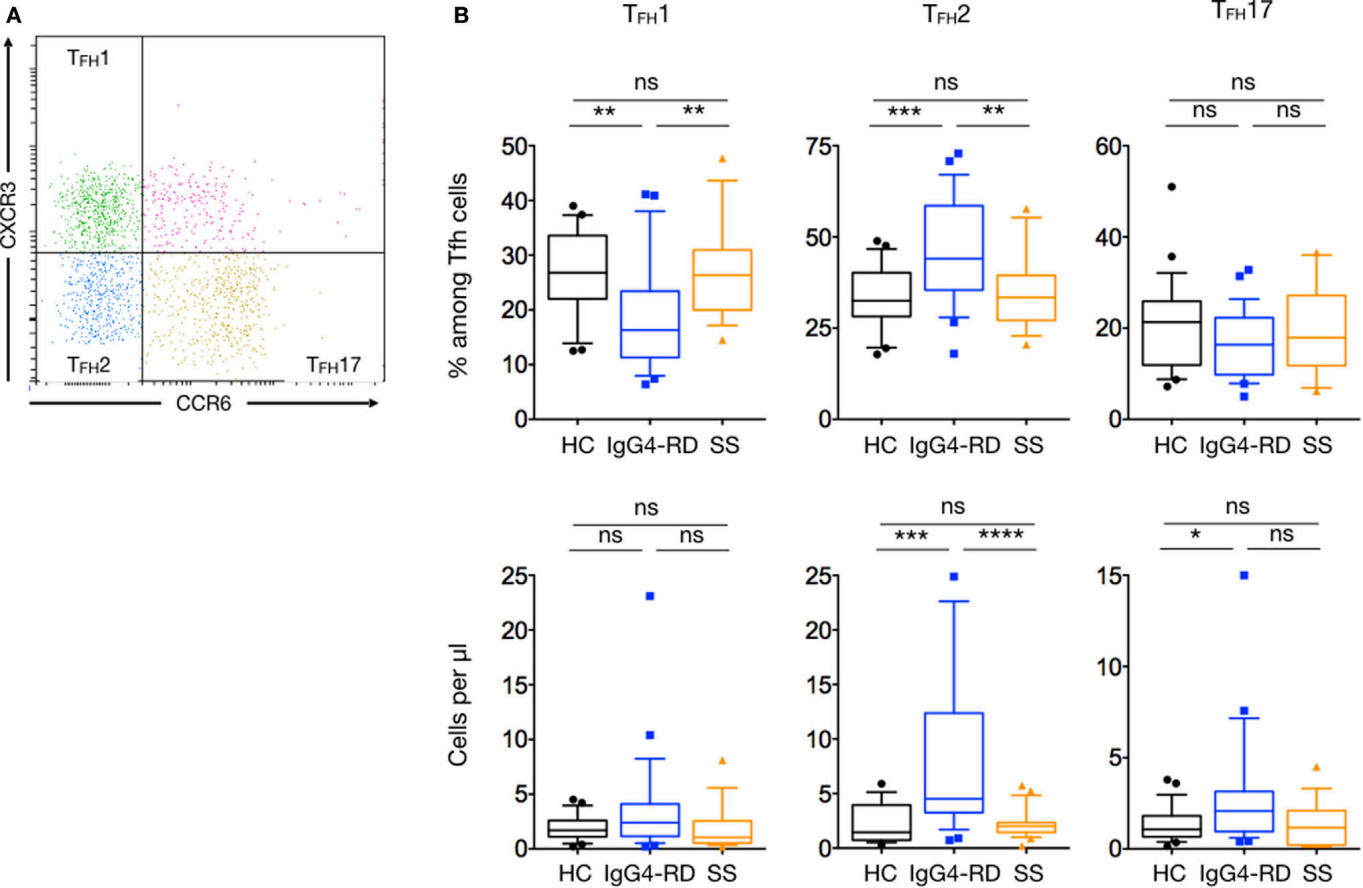
**Specific changes of T follicular helper subsets in IgG4-related disease (IgG4-RD) patients**. **(A)** A representative staining showing T_FH_ cells subsets defined according to the expression of CXCR3 and CCR6: T_FH_1 are CXCR3^+^CCR6^−^ cells, T_FH_2 are CXCR3^−^CCR6^−^ cells, and T_FH_17 are CXCR3^−^CCR6^+^ cells. **(B)** Percentage of T follicular helper subsets among total T_FH_ cells (upper panel) and cell numbers (lower panel) of T_FH_1, T_FH_2, and T_FH_17 in the peripheral blood are shown for IgG4-RD patients (blue lines), healthy controls (black lines), and Sjögren syndrome patients (orange lines). Bars show the median and the 10th and 90th percentiles. If the Kruskal–Wallis test is significant, groupwise comparisons are performed by the Mann–Whitney *U*-test. *p* is considered significant when <0.05; *p < 0.05; **p < 0.01; ***p < 0.001; ****p < 0.0001.

As for T helper cells, T_FH_ cells can be further classified into distinct subsets according to cell-surface chemokine receptor expression: T_FH_1 (CCR6^−^CXCR3^+^), T_FH_2 (CCR6^−^CXCR3^−^), and T_FH_17 (CCR6^+^CXCR3^−^) (Figure 5A). Among the CD4^+^CXCR5^+^PD1^+^ T_FH_ cells, T_FH_2 cells were the main increased subset in patients with IgG4-RD compared to HC and pSS patients for both number and percentages (8.5 ± 1.6 cells/μl versus 2.2 ± 0.2 and 2.2 ± 0.4 cells/μl; *p* < 0.0001 and *p* = 0.0004, respectively; and 44.2 ± 2.7% versus 33.2 ± 1.6 and 35 ± 2.6%; *p* = 0.0003 and *p* = 0.005, respectively). The number of T_FH_17 cells was higher in patients with IgG4-RD as compared to HC and pSS patients (2.83 ± 0.6 cells/μl versus 1.4 ± 0.1 and 1.3 ± 0.3 cells/μl; *p* = 0.03 and *p* = 0.06, respectively). Last, the proportion of T_FH_1 cells was decreased in patients with IgG4-RD as compared with HC and pSS patients (19 ± 1.9 cells/μl versus 26.6 ± 1.4 and 27.2 ± 2.1 cells/μl; *p* = 0.002 and *p* = 0.003, respectively) (Figure 5B).

## Discussion

IgG4-related disease is characterized by tissue infiltration by numerous IgG4^+^ plasmocytes, increased numbers of circulating plasmablasts and elevated titers of both serum IgG4, IgE, as well as other IgG subclasses (2). B cell activation appears to be T cell-dependent because activated B cells harbor enhanced somatic mutation, and relapses after rituximab (a B-cell-depleting agent) are characterized by the emergence of new plasmablast clones (17). T CD4^+^ cells are numerous in disease infiltrates and are thought to trigger B cell polyclonal expansion and fibrosis. Prior studies regarding blood and/or tissue analysis of IgG4-RD patients suggest changes of T helper cells, Treg, and more recently oligoclonal CD4^+^ effector/memory cytotoxic T lymphocytes (9–11, 18, 19).

Hence, we conducted a systematic analysis of lymphocyte subsets in untreated patients with IgG4-RD. Major T cell subsets, including memory and naïve T cells, NK, and B cells were normal in patients with IgG4-RD compared to HC. Concordant with previous reports, plasmablast cell counts were higher in patients with IgG4-RD as compared to HC (17). Albeit not reaching significance, plasmablast cells were also higher in patients with IgG4-RD as compared to pSS patients. Next, in line with a previous study, there was a clear trend toward an increase of circulating CD4^+^Foxp3^+^ Tregs in IgG4-RD patients, suggesting that in such patients Treg expansion is not restricted to diseased tissues (18). Treg were also shown to be increased in blood, without reaching significance, in an independent study (19). In IgG4-RD, Treg expansion might contribute to the genesis of fibrosis through TGF-β release and to the increase of IL-10 release by PBMCs. From that perspective, IgG4-RD is different than most autoimmune diseases where Treg are commonly decreased (20).

Over-production of T_H_2-related cytokines (IL-4, IL-13, and IL-5) in IgG4-RD tissues has previously been reported (11) and correlates with IgE and IgG4 class switch, blood eosinophilia, and eosinophilic infiltrates that are common features of IgG4-RD (4, 11). Hence, and despite previous studies that provided conflicting results regarding the T_H_1/T_H_2 balance in PBMCs from IgG4-RD patients, it has been suggested that T_H_2 cells might be key players in the disease’s pathophysiology (9, 10). Yet, the central role of such cells is debated because IL-4 production upon re-stimulation has been shown to be restricted to CD4^+^GATA3^+^ T cells in patients with known atopy, suggesting that the role of T_H_2 responses in IgG4-RD might be confounded by concomitant allergic disease (21). The present analysis of T_H_1, T_H_2, and T_H_17 cells in IgG4-RD showed an increase of both T_H_2 and T_H_17 cells. Interestingly, the increase of T_H_2 cells in the present study was not restricted to a unique organ involvement. Moreover, only 4 out of 28 patients (16%) with IgG4-RD also complied with the definition of the European Academy of Allergy and Clinical Immunology for atopy (22). T_H_2 and T_FH_2 cell number, IgE levels, and eosinophil numbers were not significantly different between atopic and non-atopic patients (data not shown). Hence, according to our data, T_H_2 cell expansion in IgG4-RD patients cannot be restricted only to patients with concomitant atopy. Next, the analysis of cytokine release by PBMCs upon stimulation were in line with these findings and showed that patients with IgG4-RD produced higher amounts of IL-4 but also of IL-10 and IL-17 as compared with HC and patients with pSS. Both T_H_2 and Treg are thought to contribute to IL-4 and IL-10 release, but we did not expect to report on both high IL-17 levels and elevated T_H_17 cells. Analysis of the co-expression of intracellular IL-17 and IL-4 after stimulation in CD4^+^T cells showed that double positive cells were extremely rare, and therefore that the source of both cytokines were differentiated T_H_2 or T_H_17 cells (data not shown).

Extranodal germinal centers are often found in IgG4-RD tissues, and IgG4^+^ plasmablasts harbor somatic hypermutation (17), a process occurring during B cell differentiation in germinal centers. Because T_FH_ are essential for germinal center formation and Ig class switch in humans and mice, they could be implicated in the pathogenesis of IgG4-RD (23). The circulating T_FH_ compartment in human is heterogeneous based on PD1, CCR6, and CXCR3 expression (23, 24). Here, we show that the CD4^+^CXCR5^+^PD1^+^ T_FH_ cells are specifically expanded in IgG4-RD. Interestingly, since only one patient presented with an increase of T_FH_ after treatment with rituximab, expansion is reversible in patients with IgG4-RD that are efficiently treated. Expanded PD1^+^ T_FH_ have been previously reported in human autoimmune diseases such as systemic lupus erythematosus, pSS, and juvenile dermatomyositis (25, 26). In the latter, both T_FH_2 and T_FH_17 are increased and correlate with plasmablast numbers and disease activity (26). In our analysis, CXCR5^+^PD1^+^ T_FH_ cells were not expanded in untreated pSS patients but were expanded in IgG4-RD. T_FH_ expansion was mostly related to the T_FH_2 CCR6^−^CXCR3^−^ subset and to a lesser extent to the T_FH_17 CCR6^+^CXCR3^−^ subset. T_FH_2 and T_FH_17 cells have been shown to efficiently induce *in vitro* naïve B cells to proliferate and differentiate into plasmablasts and produce all IgG subclasses, in contrast to T_FH_ 1 (25). T_FH_2 specifically produces IL-4, IL-5, and IL-13, which are important cytokines for the class switching to IgE and IgG4. The expansion of T_FH_ 2 is consistent with pathological and biological abnormalities reported in IgG4-RD patients. Our study showed that T_FH_2 cell numbers correlated positively with serum IgG4 (*r* = 0.64; *p* = 0.0004), IL-4 (*r* = 0.55; *p* = 0.01), and IL-10 (*r* = 0.49; *p* = 0.03) (Table 3). Moreover, an increase of the CD4^+^CXCR5^+^CD45RA^−^ T_FH_ and T_FH_ 2 cells in IgG4-RD has been reported in another series of 15 patients (14). However, PD1 expression was not analyzed. The specific expansion of CXCR5^+^PD1^+^ T_FH_ observed in our study could be related to some unique functional properties inherent to IgG4-RD’s pathogenesis. Indeed, PD1^+^ T_FH_ require less activation than PD1^−^ T_FH_ to differentiate into functional helpers and, by opposition to PD1^−^ T_FH_, PD1^+^ T_FH_ express low levels of CCR7 (24). The PD1^+^CCR7^low^ T_FH_ population is required for T cells to migrate into B cell follicles (27). Thus the specific expansion of PD1^+^ T_FH_ in IgG4-RD could be an important trigger to B cell activation, class switch, and plasmablast generation. Interestingly, it has been shown in rheumatoid arthritis that PD1^+^ T_FH_ is maintained by plasmablasts by an IL-6-dependent positive feedback loop that should be investigated in IgG4-RD (28).

**Table 3:** **Analysis of the correlation between T_FH_ and T_FH_2 cell number and clinical or biological variables in patients with IgG4-RD**.

Correlation between variables	*r* (Spearman)	*p*-Value	Adjusted *p*-value
T_FH_ and IgG4-RI	−0.15	0.52	0.71
T_FH_2 and IgG4-RI	0.2	0.36	0.62
T_FH_ and number of OI	−0.03	0.85	0.86
T_FH_2 and number of OI	0.14	0.48	0.70
T_FH_ and IgG4	0.3	0.13	0.35
T_FH_2 and IgG4	**0.64**	**0.0004**	**0.008**
T_FH_2 and IgG1	0.21	0.31	0.62
T_FH_2 and eosinophils	−0.04	0.86	0.86
T_FH_2 and IgE	0.24	0.33	0.62
T_FH_ and plasmablast	−0.03	0.20	0.48
T_FH_2 and plasmablast	0.37	0.1	0.32
T_FH_ and IL-10	0.18	0.47	0.70
T_FH_2 and IL-10	**0.49**	**0.03**	0.14
T_FH_ and IL-4	0.06	0.8	0.86
T_FH_2 and IL-4	**0.55**	**0.01**	0.09
T_FH_ and IL-17	0.41	0.1	0.32
T_FH_17 and IL-17	**0.62**	**0.004**	**0.04**
T_FH_2 and IL-17	0.08	0.72	0.86
T_FH_1 and IFNγ	0.12	0.62	0.79

*The cytokines listed in the variables column correspond to cytokines released from peripheral blood mononuclear cells following 24 h stimulation with PMA-ionomycin and measured using a multiplexed CBA assay*.

*Correlations were performed by analyzing T_FH_ cell number/μl with other variables. *p* was considered significant by Spearman test when <0.05 (in bold). For multiple testing, adjusted *p*-values were calculated using the false discovery rate procedure*.

*IgG4-RI, IgG4 responder index; OI, organ involvement*.

The findings reported in our study consist of correlations and causation of these T cells changes in the pathophysiology of IgG4-RD have to be confirmed by further functional studies. It has been recently shown in Japanese patients with predominant salivary and lachrymal glands involvement that CD4^+^CD45RA^−^CXCR5^+^CCR6^−^CXCR3^−^ T_FH_2 cells were more efficient in inducing differentiation into plasmablasts and led to higher IgG4 production by autologous naïve B cells in active, untreated IgG4-RD patients than in HC (29), suggesting a functional role of these cells in the disease. The same authors found in a previous study a correlation between the increased number of circulating T_FH_2 cells and the number of plasmablasts (14), which was not found in our study. Conversely it is also plausible that these T cells changes are secondary to yet other unknown factor(s) (e.g., a source of TGF-β) that drives T cell differentiation and IgG4 production. In this line, mast cells have recently been shown to express IL-4, IL-10, and TGF-β (30), as well as IL-13 (31) in IgG4-RD tissues, and these innate cells could contribute to the T_H_2/T regulatory cytokines orientation reported in the disease.

Major cytokines involved in the early T_FH_ differentiation process from CD4^+^ T cells in human, including IL-12, IL-23, and TGF-β, are also supported by other STAT3-activating cytokines including IL-6, IL-21, and IL1-β (23). In human autoimmune diseases, both T_H_17 and T_FH_ co-emerge and share a developmental mechanism induced by TGF-β. It has been proposed that abundant expression of TGF-β in inflammatory sites in human autoimmune diseases (28), where tertiary lymphoid organs are often formed, contribute to the generation of T_FH_ and T_H_17 cells (24). Hence, the expansion of these cells could be the consequence of an initial inflammatory process. In tissues, T_H_17-related molecules have been reported in salivary glands of patients with IgG4-RD, albeit at low levels (32). The site where the differentiation and expansion of T_FH_ occurs in IgG4-RD is unknown, and no link has been established with IL-1β and TGF-β producing clonal expanded CD4^+^SLAMF7^+^ CTLs (19).

Interestingly, our results showed that PD1^+^ T_FH_ cells were significantly decreased in patients with IgG4-RD who were treated with either steroids alone, steroids plus azathioprine, or steroids plus rituximab, and that the decrease in PD1^+^ T_FH_ cells was always associated with clinical improvement of the disease. Significant variation of T_FH_ cells after treatment with steroids has also been recently reported in Japanese patients with modification of T_FH_1 cells, which we did not observe in our larger study (29). However, organ involvement was significantly different from our study. Interestingly, the PD1^+^ T_FH_ subset also comprises T_FH_ regulatory cells co-expressing Foxp3 and an imbalance of T_FH/_T_FH_ Foxp3 regulatory could also be implicated in the disease’s pathophysiology and should be further explored (33).

## Conclusion

We show that patients with active, untreated IgG4-RD presenting with variable organ involvements present specific changes of T cells in peripheral blood. Circulating PD1^+^ T_FH_ are expanded together with T_H_2 and T_H_17, associated with elevated IL-4, IL-10, and IL-17 release. Both PD1^+^ Tfh2 and Tfh17 subsets have been shown to be prone to migrate into B cell follicles and/or inflammatory sites and could therefore contribute to B cell activation and IgG4 class switch in IgG4-RD tissues. Yet, the precise role of these T cell changes together with the role of clonal expanded cytotoxic T cells, their interactions with B cells and other immune cells, and the induction of fibrosis remains to be further assessed in IgG4-RD. Indeed, in a near future, it is likely that specific treatments targeting T cells will be assessed in IgG4-RD.

## Author Contributions

AG, ME, FV, and NS: conception and design of the work; acquisition, analysis, and interpretation of data for the work; drafting the work and revising it critically for important intellectual content. CP, CF, J-RH, and NC-C: acquisition, analysis, and interpretation of data for the work; revising the work critically for important intellectual content. MG, AR, MS, BT, NM, SA, FM, JG, MH, AF, SP, EB, and BB: acquisition of data for the work; revising the work critically for important intellectual content. All authors meet following criteria: final approval of the version to be published and agreement to be accountable for all aspects of the work in ensuring that questions related to the accuracy or integrity of any part of the work are appropriately investigated and resolved.

## Conflict of Interest Statement

The authors declare that the research was conducted in the absence of any commercial or financial relationships that could be construed as a potential conflict of interest.
