# Supplementary figures and images for: T Cell Polarization toward TH2/TFH2 and TH17/TFH17 in Patients with IgG4-Related Disease
Source: Front Immunol. 2017 Mar 13;8:235. doi: 10.3389/fimmu.2017.00235 (PMC5347096; doi:10.3389/fimmu.2017.00235)

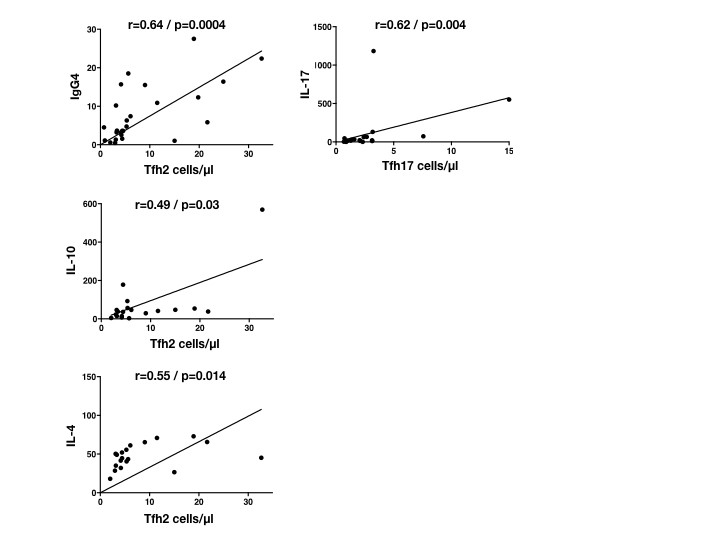

Supplement: Supplementary file 1 [file Image_1.JPEG]
